# P34 A case of Entamoeba histolytica infection in a patient with polymyalgic-onset inflammatory arthritis

**DOI:** 10.1093/rap/rkac067.034

**Published:** 2022-09-28

**Authors:** Sabreen Ali, Dana Hassan-Bendahan, Anne Kinderlerer

**Affiliations:** St Mary's Hospital, Imperial College NHS Healthcare Trust, London, United Kingdom; St Mary's Hospital, Imperial College NHS Healthcare Trust, London, United Kingdom; St Mary's Hospital, Imperial College NHS Healthcare Trust, London, United Kingdom

## Abstract

**Introduction/Background:**

Reactive and post-infectious arthritides can be challenging to diagnose and manage. Establishing a causative link between pathogen, if indeed one is identified, and subsequent rheumatological symptoms can be difficult. Infection may mimic rheumatic disease, exacerbate it and has consequences for treatment decisions as well. Increasing globalisation has lead to the spread of disease from endemic to non-endemic regions, making this issue ever more relevant. We present the case of a patient with inflammatory symptoms ultimately diagnosed with Entamoeba histolytica parasitic infection.

**Description/Method:**

A 56-year-old Italian restaurant manager developed acute bilateral hip pain soon after his first coronavirus vaccination in June 2021. Prior to the joint symptoms he reported an episode of conjunctivitis, as well as right testicular pain which was treated as epididymo-orchitis. He had a past medical history of hypertension and asthma.

He developed progressive stiffness and pain affecting his shoulders and arms, and was commenced on 15mg of prednisolone for suspected polymyalgia rheumatica (PMR) in the context of raised inflammatory markers.

On review in our department, the patient reported very limited response to steroid therapy. He had also developed vomiting, diarrhoea, fever, night sweats and weight loss, exacerbated when weaning prednisolone. These symptoms persisted despite increasing the dose to 20mg. He recalled having a few months of diarrhoea prior to his presentation, up to 5 times a day.

Examination did not reveal synovitis or muscle weakness; cardiopulmonary and abdominal examination were normal with no palpable lymphadenopathy. CRP was persistently elevated at 38 mg/L. Serology was negative for ANA, ANCA, CCP, RhF and HLA B27. HIV and TB Elispot were negative, with evidence of previous hepatitis C infection. Urinalysis was negative for haematoproteinuria.

PET scan showed colonic inflammation suggestive of ascending colitis with ileocolic lymphadenopathy. Subsequent colonoscopy demonstrated patchy macroscopic inflammation; biopsies were positive for Entamoeba histolytica infection.

Suspected method of acquisition was by ingesting unwashed tropical fruits, which he consumed frequently. He was treated with Metronidazole and Paromomycin with rapid resolution of his joint and constitutional symptoms. He was able to successfully wean steroids for the first time since feeling unwell, however began developing peripheral joint pain with bilateral metacarpophalangeal joint synovitis evident on clinical examination on weaning to 2mg of prednisolone. He was commenced on Methotrexate, with follow-up due in the near future.

**Discussion/Results:**

90% of patients with Entamoeba histolytica amoebiasis are asymptomatic, however some will present with a subacute colitis. Recent reports have identified this parasite as a rare potential cause for reactive arthritis, which is the likely scenario in this case. Our patient developed conjunctivitis and genito-urinary symptoms (usually urethritis but epididymitis has been reported) in conjunction with joint symptoms, as is classically described.

The patient had significant diarrhoea and vomiting throughout his illness; the former pre-dated steroid therapy, although it is possible that steroids exacerbated his symptoms. Prompt resolution of his joint pain and stiffness upon treatment of the parasitic infection is also strongly suggestive of a link between the two, and is unlikely to have occurred if the Entamoeba was purely incidental.

He may have gone on to develop a chronic reactive arthritis given the occurrence of peripheral inflammation on lower doses of prednisolone, now requiring disease modifying therapy.

Alternatively, the patient may have developed a polymyalgic onset of inflammatory arthritis with incidental Entamoeba infection, however given the reasons above and atypical steroid response this is thought to be less likely. We also considered the possibility of polyarteritis nodosa (PAN) given prominent abdominal symptoms and testicular involvement at onset, however he had no aneurysmal disease on imaging and no other specific features of this condition.

**Key learning points/Conclusion:**

Literature review suggests that arthritis induced by parasitic infestation is polymorphic and heterogenous, and is likely to involve genetic predisposition in terms of patients who ultimately develop rheumatological sequelae. Minimal response to classical anti-rheumatic agents, including steroids and anti-inflammatories with subsequent profound improvement secondary to anti-parasitic treatment is an important clue to the diagnosis.

A variety of microbes can trigger HLA B27 non-associated reactive arthritis, in which a polyarthritis is more common compared to oligoarthritis. Interestingly in a series of cases of parasitic infection with rheumatic manifestations, HLA B27 was negative in 9 out of 13 patients, of whom 7 had a symmetrical polyarthritis.

A recent Egyptian study analysed stool samples from 107 patients with unexplained rheumatic pain - 50 had parasitic infection. Parasitic rheumatism was ultimately diagnosed in 16 of these patients, with Entamoeba histolytica found in 8% of cases; around a third of these patients had resolution of their symptoms after anti-parasitic therapy.

It remains important, however, to avoid over-attributing pathology to reactive phenomena and alternative diagnoses such as malignancy and vasculitides should always be considered.

Our patient had not travelled abroad for 5 years and a lack of travel history may distract from parasitic disease as a diagnosis; nevertheless increasing global connectivity and emigration means that non-endemic infection is rising in developed countries. More detailed evaluation of his history may have highlighted symptoms of diarrhoea and prompted stool culture and colonoscopy prior to steroid therapy, potentially leading to an earlier diagnosis.

Overall, this case highlights the importance of accurate diagnosis in patients who present atypically, and re-evaluation of patients who do not respond to treatment as expected. Infectious diseases remain a vital consideration in the assessment of patients presenting with systemic symptoms, and histopathological assessment should be sought were possible.

